# Multi-LiDAR Mapping for Scene Segmentation in Indoor Environments for Mobile Robots

**DOI:** 10.3390/s22103690

**Published:** 2022-05-12

**Authors:** Pavel Gonzalez, Alicia Mora, Santiago Garrido, Ramon Barber, Luis Moreno

**Affiliations:** Robotics Lab, Universidad Carlos III de Madrid, 28911 Leganes, Spain; sgarrido@ing.uc3m.es (S.G.); rbarber@ing.uc3m.es (R.B.); moreno@ing.uc3m.es (L.M.)

**Keywords:** LiDAR odometry, scan matching, SLAM, scene segmentation, topological, Harmony Search

## Abstract

Nowadays, most mobile robot applications use two-dimensional LiDAR for indoor mapping, navigation, and low-level scene segmentation. However, single data type maps are not enough in a six degree of freedom world. Multi-LiDAR sensor fusion increments the capability of robots to map on different levels the surrounding environment. It exploits the benefits of several data types, counteracting the cons of each of the sensors. This research introduces several techniques to achieve mapping and navigation through indoor environments. First, a scan matching algorithm based on ICP with distance threshold association counter is used as a multi-objective-like fitness function. Then, with Harmony Search, results are optimized without any previous initial guess or odometry. A global map is then built during SLAM, reducing the accumulated error and demonstrating better results than solo odometry LiDAR matching. As a novelty, both algorithms are implemented in 2D and 3D mapping, overlapping the resulting maps to fuse geometrical information at different heights. Finally, a room segmentation procedure is proposed by analyzing this information, avoiding occlusions that appear in 2D maps, and proving the benefits by implementing a door recognition system. Experiments are conducted in both simulated and real scenarios, proving the performance of the proposed algorithms.

## 1. Introduction

Mobile robot applications in indoor scenarios have been rapidly increasing in the last decades thanks to the advances of new sensor technologies such as LiDARs and depth cameras. Because of these sensors’ decreasing prices, it is now easy to find low-budget robots with tiny sensors to save environment information and perform tasks such as processing maps, localizing themselves, and sometimes planning simple paths to move to a specified target. However, most high-level applications with bigger robots require more complex capabilities, such as manipulating. It implies navigating around the environment and achieving multiple tasks from the starting point to the goal area, such as calculating the configuration of one or two arms. Additionally, these robots are deployed in environments designed for people. It means that, apart from geometric characteristics, higher-level information with a semantic meaning needs to be measured and analyzed. For instance, by detecting how indoor environments are arranged into different rooms, navigation can be made easier, getting closer to how people interpret their surroundings. For that reason, it is essential to exploit the uses of environment information coming from diverse sensor sources for achieving a robust indoor representation. Other works have already proven the importance of using diverse sensor sources for overcoming problems such as precise geometric localization [1] or mapping problematic regions with glass surfaces [2]. However, previous reviewed papers have stated that sensor fusion using LiDAR and camera sensors is very sensitive to daylight because it interferes with the IR light. In addition, algorithms based on RGBD are likely to misalign in corridors that are untextured and seem alike in consecutive poses. For the past five years, LiDAR SLAM-based algorithms have implemented LiDAR fusion with IMU, and there have not been significant changes in these approaches. They try to correct motion using IMU velocity information [3]. This work proposes a Multi-LiDAR mapping method based on 2D and 3D sensors at different heights. The aim is to create a map system with both information types adequately aligned to provide environment information at different heights, becoming available for a wide variety of robot applications such as navigation or manipulation. As an example of the use of information coming at different heights from the resulting map, an indoor segmentation procedure is proposed to extract topological information from a scenario. 2D navigation and segmentation of indoor ambiance is a fast-growing research topic. Typical works perform algorithms based on information retrieved from a single LiDAR sensor positioned at the robot base, slightly displaced above ground height. Once distance information is available, a 2D map is built using SLAM techniques. Finally, its geometric characteristics are analyzed to divide the map into meaningful regions. Still and all, real-life applications at a low height always show many occlusions, obstacles, and objects on the ground that make it difficult to get a well-known segmented map. As a second goal to be achieved, geometric characteristics at different heights on the map are analyzed, proving the necessity and benefits of using and fusing multiple types of dense data to improve scene segmentation and further path planning.

**Figure 1:**
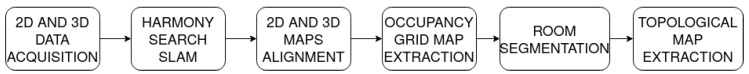
Proposed steps for creating a Multi-LiDAR mapping system and extracting a topological map afterwards.

Figure 1 shows the proposed steps to create a mapping system using Multi-LiDAR sensor fusion in order to obtain environment information at multiple heights. This information is then used to segment an indoor scenario to obtain topological information. Initially, environment data are acquired using 2D and 3D LiDAR sensors. Then, Harmony Search SLAM aligns captured scans, deriving into the next step, which is aligning 2D and 3D maps. These maps are then transformed into an occupancy grid map, which is used to geometrically segment the scenario into rooms and finally to extract a topological map.

These techniques will be explained in more detail in the following sections. The rest of the work is divided as follows. Section 2 reviews state of the art for LiDAR mapping and room segmentation. Section 3 describes the method used for mapping. Section 4 proposes a method for room segmentation. In Section 5 materials and methods used are described. Results are shown in Section 6 and discussed in Section 7. Finally, conclusions are provided in Section 8.

## 2. Related Work

This section presents related work regarding map acquisition and scene segmentation. Initially, techniques for obtaining a two and three-dimensional geometric map are reviewed. Then, room segmentation procedures are analyzed, given that they rely on a previously acquired map using the above-mentioned techniques. Finally, optimization procedures for scan matching are shown.

### 2.1. Simultaneous Localization and Mapping

Simple categorization states there are two map types, geometrical and topological. In order to create a topological map, most applications start by knowing or building a previous map, typically a geometrical map with distance information of the environment such as walls, furniture, and objects. These maps depend on the sensor used to capture the scene, usually a 2D sensor parallel to the floor and perpendicular to the environment or a 3D sensor that can measure volumetrically by pointing in different directions at a time. 2D fixed LiDAR sensors on mobile platforms generate 2D geometrical maps and a 3D pose of the robot given by the displacement on *x*, *y*, and rotation rz around the *z* axis of the robot. For 3D fixed sensors, the pose vector expands to a six degrees of freedom problem, where the pose of the robot may change in three-axis *x*, *y*, *z* and three rotations rx, ry, rz, around each axis or roll, pitch, yaw angles.

In pursuance of building a consistent and well-defined map, many authors since the early 90s have proposed plenty of techniques to achieve a successful registration between data scans. There are three main widely spread algorithms that have been the foundations of many implementations and variations through the years. Iterative Closest Point ICP [4] introduces one of the most used and modified algorithms. Its goal is simple yet effective: to calculate a rotation and a translation (R,t) by minimizing the least-squares of the distance of all the points that belong to two different and consecutive observations defined by scans *A* and *B*, that yields the minimum possible distances between them.

Equation (1) represents the classical fitness function of the ICP algorithm, where the error *E* of a rotation *R* and a translation *t* is the summation of all the euclidean distances between two scans or point clouds *A* and *B* with Na and Nb number of representative points.

(1) $$E\left(R,t\right)=\sum_{i=1}^{Na}\sum_{j=1}^{Nb}{∥a_{i}-\left({\mathrm{Rb}}_{j}+t\right)∥}^{2}$$

**Figure 2:**
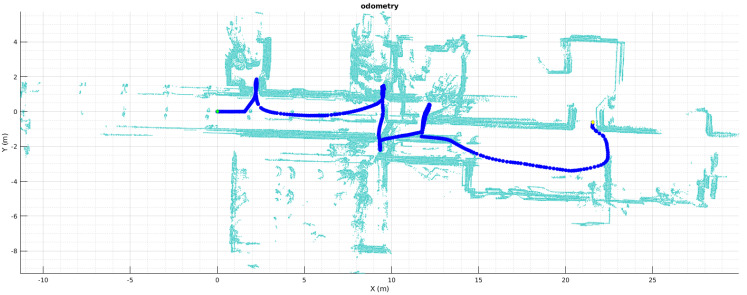
Map built based on robot odometer.

Lu and Milos [5] solved the same problem based on the maximum likelihood criterion to optimally combine all the spatial relations and use those as constraints for the data frame poses. Last, the Normal Distribution Transform approach [6] states that a scan can be subdivided into piece-wise continuous and differentiable probability density and uses this information to match successive scans using Newton’s algorithm. One of the primary purposes of building a good consistent map is to be able to transform the scans or point cloud into another type of data such as an occupancy grid to perform path planning and navigation. Figure 2 shows how a bad set of mismatching scans throws a map with a lot of misaligned points and an impossible robot path and location, demanding a correction on both the mapping and localization field. This map building does not provide good data for further analysis and segmentation. In addition, this data type is not the same needed to perform path planning and segmentation, indicating that an occupancy grid based on geometrical information is required at a second stage to generate a topological map based on room segmentation.

### 2.2. Room Segmentation

Occupancy grid maps are traditionally partitioned by analyzing their geometric properties. Some works propose the extraction of critical points to define partitions [7,8,9]. Additional techniques are applied afterwards to remove unnecessary cuts. Another method is Watershed algorithm [10,11,12], in which heuristics are also needed to merge regions, since oversegmentation occurs due to local minima. Voronoi graphs are one of the most commonly used techniques, in which their structure is analyzed to detect critical regions [13,14] or they are combined with other techniques such as morphological operations [15] or alpha-shapes [16]. This method has been proven to outperform other segmentation procedures [17]. Other segmentation methods rely on learning techniques [18,19,20,21], so despite the fact that they are effective, their design is complex and computationally expensive since they require training procedures.

The 3D representations are used to benefit from its prior analysis before generating a 2D partitioning. Some works propose the analysis and projection of 3D data onto the X-Y plane to create an occupancy grid map [22,23]. In [24,25,26], only vertical planes corresponding to walls are detected and projected. Treating with big quantities of 3D data cause these methods to be computationally expensive. The required time to partition data into planes and afterwards compute wall segments is high and becomes increasingly higher for denser point clouds. In this work, we propose the use of a slice of a point cloud to reduce computational costs. Moreover, it is chosen so that furniture is avoided, leading to better segmentation results.

### 2.3. Non-Linear Optimization

To achieve a good rigid registration, segmentation, or scene recognition, several mathematical approaches should be stated and solved. Room segmentation in this research is based on solving linear equations in a stochastic way but this is not the case to solve the registration part of the SLAM algorithm for both 2D and 3D. Scan matching can be reduced to an optimization problem, so many authors use classical optimization algorithms to minimize or maximize a fitness function. Some use Newton’s Optimization Algorithms, others use Singular Value Decomposition, among others classical optimization techniques [27], these solutions usually solve large and complex matrices with first and second derivatives. Often, a big flaw of those optimization techniques is that they rely on the assumption of a good initial state such as good odometry or state prediction. Nevertheless, usually, if the initial value is not good enough the solution falls inside local minima and the optimization is bad.

Nowadays, the use of evolutive algorithms is well-known for solving engineering problems for over four decades. In this research, the implementation of a meta-heuristic evolutive algorithm is proposed and introduced to find the best pose vector $\vec{p}$ with translation and rotation (R,t) candidate that yields the minimal distance between consecutive points clouds for the minimum error in Equation (1). Harmony Search has been chosen for this goal.

**Figure 3:**
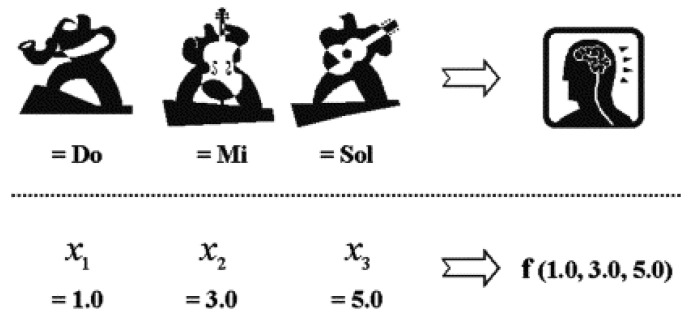
Analogy between music improvisation and engineering optimization [28].

Geem et al. [28] proposed a meta-heuristic technique inspired by Jazz musicians improvisation to find a good harmony through several iterations where every musicians represent a variable change and adjust its value to refine a good tone for a good harmony, if this harmony is better than one of the previously tried, then it is store in the group memory (See Figure 3). This meta-heuristic approach turns interesting for current research as it is flexible enough to implement opposite to Random Search or more complex meta-heuristic-like simulated annealing, or Bayesian Optimization Algorithm that have more variables for tuning in [29]. The next section explains the implementation of HS for solving the scan registration.

## 3. SLAM Based on Harmony Search

### 3.1. LiDAR Odometry

LiDAR Odometry is a technique needed for mapping environments where several issues may occur and the robot odometry cannot be trusted. Problems such as sensor accuracy, slipping floors, traction loss, among others, lead to mismatch a good result of a map based on only pure robot wheel odometry or sensor fusion with IMUs, trowing inconsistent matching, and low-quality maps. These issues may happen randomly and the uncertainty is high and difficult to calculate when the map built rely only on relative sensor information, hence it demands the implementation of an accurate LiDAR Odometry algorithm that finds the relation between poses. Figure 2 shows a map built using the odometry information provided by the robot and a 2D Hokuyo LiDAR. Laser scans are shown in light blue, whereas the dark blue line appears as sensor poses. It can be noticed that mismatching of the scans results in thicker walls in corridors and incomprehensible doors, causing this map to be useless for localization, segmentation, path planning, or navigation.

In previous research [30], several tests and developments of scan matching algorithms based on evolutive algorithms such as Differential Evolution (DE) have been tried and introduced. Now, this development extends those algorithms in 2D and 3D rigid registration based on a mixed weighting function, similar to a multi-objective optimization, using Harmony Search as the optimization algorithm. The main goal of the scan matching algorithm is to find a pose vector $\vec{p}$ containing the 3DOF (x,y,rz) for 2D maps that achieves the best registration possible of two scans or point clouds *A* and *B* that represents a good scene also a correction of the odometry pose.

### 3.2. Full 6DOF Harmony Search SLAM

Typically the next extension to a single rigid registration of two consecutive scans representing a slice of the environment around the robot is the simultaneous localization and mapping of several consecutive poses of the robot trajectory. LiDAR odometry extension to SLAM algorithm rests on merging scans and poses based on the previously calculated pose $\vec{p}$. Some techniques rely on stochastic calculations, some others rely on probabilistic equations trying to assure a good registration by optimizing a single value function. The proposed algorithm is a variation of the classical ICP described in Section 2, introducing a simple but significant improvement by modifying the single fitness functions with a two value fitness function and implementing the Harmony Search optimization algorithm, plus an initial population guesses formed by previous poses and global map feedback. The fitness function in Harmony-ICP is the minimization of E(R,t) and the maximization of the number of points Nb at scan *B* that falls below a threshold of distance to scan *A*. This algorithm is feasible to unfold to 6DOF by finding a vector $\vec{p}$ with (x,y,z,rx,ry,rz). Based on the Harmony Search meta-heuristic for 2D maps, the optimization has three musicians. For 3D maps and full 6DOF, the band has six musicians, one for each variable in $\vec{p}$, searching an optimal location for all six variables at the same time. By looking at Algorithm 1, its goal is to search for a good pose by finding a good harmony inside a defined range and avoiding linear optimization issues of false convergence if the initial states where not good enough. From now on the algorithm for global mapping implementing Harmony-ICP is presented as HS-SLAM.
**Algorithm 1** Harmony-ICP**Require:** 
*A*, *B*, maxIter,$init\vec{G}uess$**Ensure:** 
$\vec{p}$(x,y,z,rx,ry,rz) $HM_{CR}\leftarrow 0.7$PAR←0.3BW←0.05$HM_{size}\leftarrow 30$trialH←[0,0,0,0,0,0]**while**N≤maxIter**do**    trialHramdom(HM)                ▹ Improvise a new Harmony    $trialH\leftarrow mix(trialH,HM,HM_{CR})$▹ Mix some instruments with HM based on CR    trialH←AdjustPitch(trialH)∗BW    $Cost_{trialH}$                    ▹ Evaluate Equation (1) for trialH    **if** $Cost_{trialH}\leq max\left(costHM\right)$ **then**        HMmax←trialH               ▹ Update the Harmony Memory    **end if**    bestHarmony←min(HM)    worstHarmony←max(HM)    **if** bestHarmony≡worstHarmony or ∀HM≡[0,0,0,0,0,0] **then**        break    **end if****end while**$\vec{p}\left(x,y,z,rx,ry,rz\right)\leftarrow BestHarmony$

**Figure 4:**
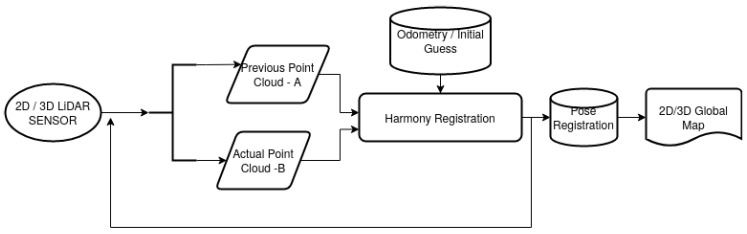
Implementation of HS-SLAM.

Figure 4 shows a flowchart of the general idea behind the SLAM process and use of several initial guess for the first population of Harmony registration. For the 3D point clouds and 6DoF registration, the information of the previously obtained 2D poses can be reuse to reduce the search range of the Harmony Search optimization. Then, after both maps are built separately, they can be aligned to apply the geometric segmentation.

## 4. Geometric Segmentation and Topological Map Extraction

Occupancy grids represent indoor scenarios with their precise coordinates. This kind of model is useful for robots to geometrically navigate. However, it is far from the way in which people interpret their surroundings. Typical indoor scenarios are divided into rooms and corridors with different utility. By analyzing geometric properties of the geometric map, it can be divided into meaningful regions. More precisely, the aim is to find separations between rooms by locating doorways or narrow passages. In this way, a step is made toward the way people understand indoor scenarios. The optimized 2D map obtained on previous steps is helpful for 2D navigation, since it contains all occupied zones that the robot cannot traverse, such as tables or chair legs. However, cluttered maps do not perform correctly regarding door detection. For that reason, we propose the use of a map obtained from a higher height, retrieved from point cloud data. In this way, we benefit from the availability of multiple information sources.

Once the occupancy grid map is available at the desired height, it is saved as a binary image for further processing, assigning value 0 for unknown and occupied spaces and value 1 for free space. The selected method for segmenting scenarios is Voronoi diagram, since it normally outperforms other segmentation procedures (see Related Work Section 2.2). First, the image is preprocessed to remove noise with the median filter, which additionally rounds map corners. This step helps to avoid the creation of unnecessary branches on the Voronoi graph. The following step is extracting the Voronoi graph from the processed map. As a simplification, a skeletonization procedure is applied, which consists on iteratively thinning free space until a structure with a single cell width is left. The skeleton is then combined with the distance transform of free space to assign distance from every cell on the graph to their closest occupied cell on the map. Those zones within a range of 40 cm ± 5 cm (half of the size for standard door width) are saved as possible door locations. The middle point of each zone is selected and a line is drawn perpendicularly until the point is connected to the two closest occupied cells in opposite directions, defining final door locations. These results are finally changed to world map coordinates. Algorithm 2 shows the above described methodology.
**Algorithm 2** Door Location**Require:** 
binGrid, resolution, Xorigin, Yorigin, doorSize**Ensure:** 
doorLocation $binGrid^{\prime }\leftarrow median\left(binGrid\right)$$skelImg\leftarrow skeleton(binGrid^{\prime })$$distImg\leftarrow distanceTransform(binGrid^{\prime })$lineSeg←skelImg·distImg**if**lineSeg(i,j)>doorSize+5cm‖lineSeg(i,j)<doorSize−5cm**then**    lineSeg(i,j)←0**end if****for** every line segment in lineSeg **do**    Find two closest pixels $\left[P_{px,1},P_{px,2}\right]$ with value 0 in binGrid to the central point of lineSeg(i) perpendicular to the orientation of lineSeg(i) in both senses.    Draw line between $P_{px,1}$ and $P_{px,2}]$ in labelIMG**end for**$P_{X(m)}=\frac{P_{X(px)}}{resolution}+Xorigin\left(m\right)$$P_{Y(m)}=\frac{rows\left(binGrid\right)-P_{Y(px)}}{resolution}+Yorigin\left(m\right)$$doorLocation\leftarrow [P_{m,1},P_{m,2}]$

A step forward can be made by extracting a topological map out of the geometrically segmented occupancy map. The segmented image obtained on the previous step is labeled and those zones with an area under 1.5 m${}^{2}$ are merged to their closest neighbor zone. It is proposed to assign a node of the topological map to every labeled zone, corresponding to rooms, and to every frontier between rooms, corresponding to doors. For the creation of room nodes, the labeled image is analyzed, checking the number of labels and saving area and centroid for each of them. With respect to door nodes, only their centroid is saved. These zones are also used to check connectivity. If two labels coincide on a door, two edges are created by joining the corresponding door centroid to the room centroids on each side. With this implementation, a topological map of the indoor environment becomes available, being useful as a complementary tool for speeding up multiple robotic applications such as path planning.

## 5. Materials and Methods

**Figure 5:**
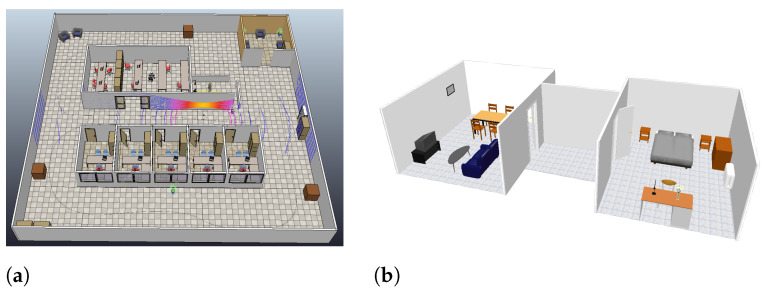
Proposed virtual scenarios. (**a**) 2D/3D SLAM. (**b**) Geometric Segmentation.

To achieve the aim and goals of this research, two approaches were implemented. The first one is based on simulated data and virtual scenarios to demonstrate the precision of the 2D/3D SLAM and the segmentation algorithms. Figure 5 shows the selected virtual scenarios for both applications. Single scan matching and HS-SLAM were tested using point clouds captured in a CoppeliaSim scene with a fixed path for a mobile robot mounting both 2D and 3D point clouds sensor, with several velocities, loops, similar areas such as corridors and turns around an indoor scene. With respect to segmentation, the scenario is formed by two rooms connected by a corridor, in which typical indoor objects such as chairs and tables are found.

**Figure 6:**
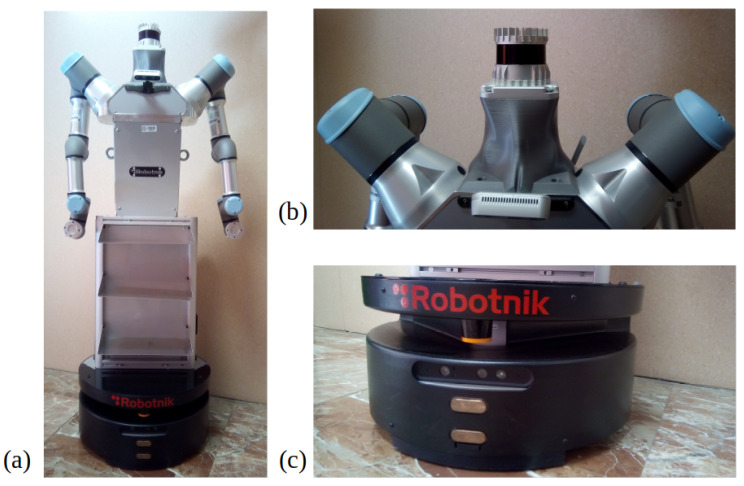
Robotic platform used for the experiments: (**a**) general robot appearance,(**b**) close-up view of the Ouster LiDAR mounted on a 3D printed neck, (**c**) close-up view of the robot base, where the Hokuyo laser is mounted (orange element).

Later, on a second stage, all these algorithms were implemented offline in real world data, captured with a mobile wheeled manipulator in differential configuration. This platform is 1.6 m tall and 60 cm in its wider size. It integrates many sensors and actuators to execute different tasks. The main one for future applications is to grasp and carry light objects from one room to another in a previously mapped and known map. The platform mounts two 6DOF arms, a 2D Hokuyo sensor with 10 m reach at 20 cm over the floor, an Asus Depth camera, differential wheels, internal IMU, on board computer and at the top of the head an Ouster 3D LiDAR of 128 channel and 50 m resolution. Figure 6 shows the platform and the sensors used for the real world data acquisition.

**Figure 7:**
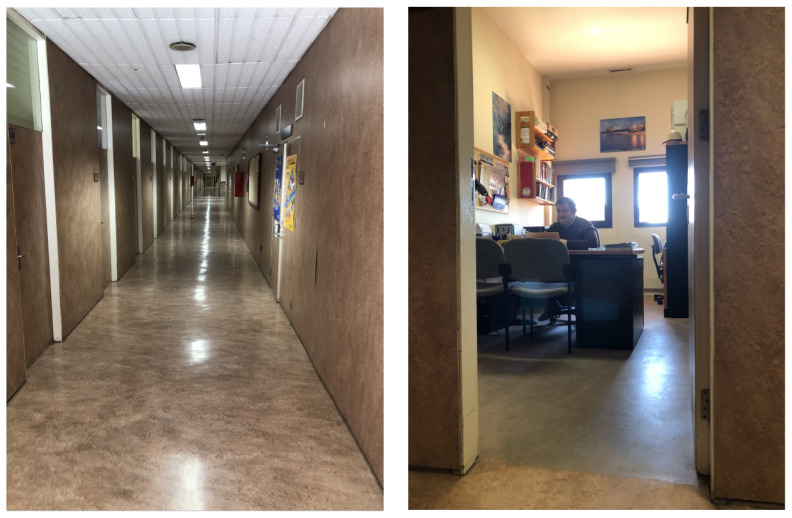
Examples of scenes on the real scenario where the mobile robot was deployed to capture 2D and 3D data: corridor (**left**) and office (**right**).

Executing a long run test, the robot was tele-operated through a hall with opened and closed doors, entered some rooms and moved to a near open area. This real world scenario has its own complexities, as the floor has no roughness and sometimes makes the wheels drift, entering rooms required a lot of rotations to both sides making odometry untrusty, and the corridor is approximately 15 m long where doors and walls look alike. An example of these facts can be seen in Figure 7, where the corridor length and its repeatibility can be observed, as well as an office cluttered with several furniture pieces such as chairs and desktops. All data were captured using ROS Melodic, saving LiDAR Scans, Odometry, Point Clouds and Timestamps. Algorithms were developed and tested in Matlab 2019b.

## 6. Results

Results presented in this part of the research are subdivided in order of implementation and importance for the research’s goal. Initially, the above mentioned virtual scenarios are considered in order to provide a first overview of results for the proposed methodologies. Then, environment information is gathered from a real scenario, which adds the difficulty of dealing with real sensor data and their corresponding measuring errors. This part starts with the 2D SLAM tests, continues with the corrections of Lidar Odometry, implements the extension of the algorithm to 3D and using the 2D pose data, follows through the segmentation of the scenario, them gathered the maps together to implement the segmentation in real data, and finally demonstrates the doors extraction to validate the research’s goals.

### 6.1. HS-SLAM

As described in Section 3, the HS-SLAM algorithm presented in Algorithm 3 is an amplification of Harmony-ICP algorithm for scan matching, adding features that improves the further registrations and the map consistency. The implementation in 2D or 3D are quite similar in code yet not in computation time. An oversimplification of the 2D algorithm is to set *z*, rx, and ry to zero, meaning no translation nor rotation in those axis. To get a global map consistency, an initial pose initPose matrix is defined with initial guess or candidate for the harmony candidates that will belong to the starting Harmony Memory, those could be the odometry, the previous pose, and mean of several previous poses, these help Harmony Search to firstly iterate around those assumption yet not being the only possible candidate for the optimal position. Fixed variable such as maxIter is set as maximum number of iteration the algorithm should internally run before stopping if the optimal value is not reach yet based on the stopping criterion, e.g., all candidates have the same values, or the difference between best and worst candidate is below a tolerance. HS-SLAM on consecutive poses and point clouds ensures a matrix *P* with all the corrected robot poses and a point cloud Map with the aligned global map.
**Algorithm 3** Harmony-SLAM**Require:** 
$M(A_{t}...A_{t+n})$**Ensure:** 
P(x,y,z,rx,ry,rz)Map maxIter←500initPose←[0,0,0,0,0,0];optional(odometry)N←2**while**N≠n**do**    **if** N≤6 **then**        $A\leftarrow M_{n-1}$                                ▹ Reference cloud    **else if** N≥6 **then**        $A\leftarrow Map_{n-1}$    **end if**    $B\leftarrow M_{(}n)$                                  ▹ Moving cloud    $\vec{p}\leftarrow HarmonyICP\left(A,B,maxIter,initPose\right)$    $P_{t}\leftarrow \vec{p}$    $initPoses\leftarrow P_{t}$    $Map_{t}\leftarrow B\left(P_{t}\right)$**end while**

The simulated scenario was used to prove the accuracy of the algorithm and the mixed fitness function. The robot went through the simulation with a total of 177 poses storing its ground truth. Position 1 was $\vec{p}$ equals all zeros, and last position was $\vec{p}$ with values (3.63 m − 4.025 m 0.0001 m 0${}^{\circ }$ 0${}^{\circ }$ 96.06${}^{\circ })$. After the whole map was reconstructed the HS-SLAM got a final position $\vec{p}$ with (3.62 m − 4.06 m 0.001 m 0${}^{\circ }$ 0${}^{\circ }$ 95.8${}^{\circ })$. This indicates that the algorithm had an error of less than 4 cm and 1${}^{\circ }$ for a rout of more than 100 m long. Table 1 summarizes the max error obtained in the worst position registered for every degree of freedom against the ground truth poses, showing that these values are inside an uncertainty range typical of LiDAR sensors plus the uncertainty drafted from the down-sampling methods.

The accumulated error at the final pose of the simulation run, and the bigger error calculated between relative LiDAR odometry poses from HS-ICP and ground truth, differ and is not the summation of all previous error thanks to the use of the global map as a feedback information to improve the registration between point cloud *A* and *B* during the pose estimation search.

#### 6.1.1. 2D LiDAR Odometry

Before applying segmentation and navigation a proper map is required, demanding the implementation of LiDAR Odometry corrections for the robot poses. Pseudo-code of Algorithms 1 and 3 recap the steps for 6DoF, instead their 2D variants were used to correct the misaligned odometry map seen in Figure 2.

**Figure 8:**
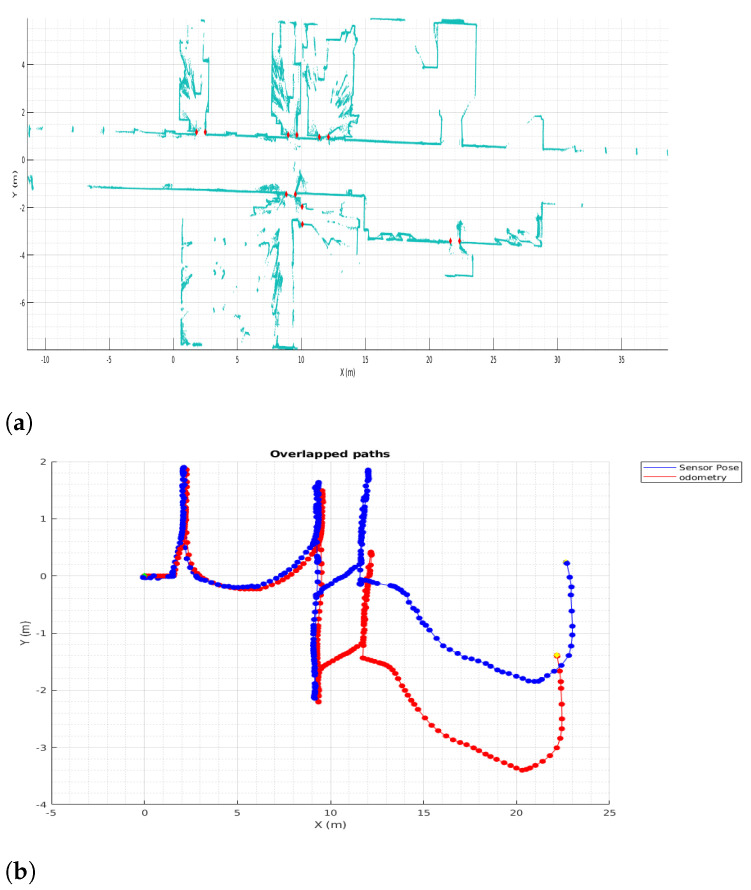
2D HS-SLAM. (**a**) 2D HS-SLAM results. (**b**) Odometry and Lidar Odometry.

Figure 8 shows, the results for 2D mapping and localization. Figure 8a demonstrate a lot of improvement against Figure 2, first, the map is consistent all the way, no thicker walls, no corridor distortion, no strange rotation, and visually the room definition is persistent. In addition, in Figure 8b dark blue line represents the LiDAR Odometry path and red line represents the odometry path same as Figure 2; here the upgrade gotten up by HS-SLAM is undeniable, specially at the center of the path where a lot of rotation and back and forth movements occurs and the mismatching of the robot odometry is bigger.

#### 6.1.2. 3D LiDAR Odometry

**Figure 9:**
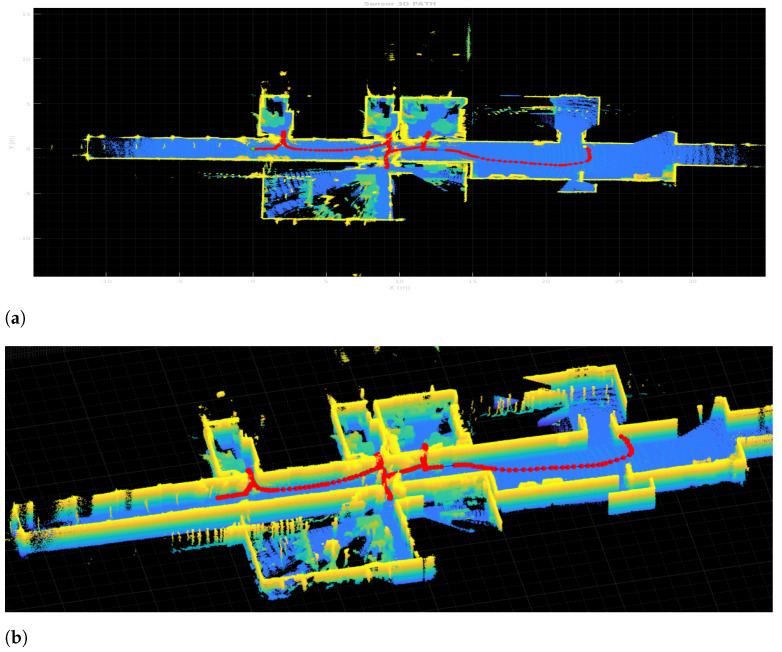
3D Harmony-SLAM. (**a**) Top view. (**b**) Side view.

The results for the full 6DoF SLAM for the 3D point clouds captured with the 3D Ouster LiDAR also expand the overall map complexity and possible non-linearity in the matching. Figure 9 shows the resulting map from pure LiDAR Odometry obtained with HS-SLAM without any robot odometry used as initial guess. Yet to help out the run time, the 3D implementation is taking advantage of the previous 2D HS-SLAM results, by using the previous 2D *P* matrix of poses for the Hokuyo LiDAR in the initial population pose matrix to enhance searching for 3D poses. Having at least an approximation of local minima for *x*, *y*, and rz weighs first this candidate during the first iterations where the exploration occurs. Figure 9a displays the top view of the whole map, ceiling have been removed to appreciate the width of the wall; Figure 9b also displays a side view zoomed in to appreciate doors and other objects in the environment

### 6.2. Geometrically Segmenting Scenarios

In this part of the work, the results of applying the proposed methodology for room segmentation is shown. Initially, a simplified virtual scenario is used for a better understanding and interpretation of the method results. Then, the same procedure is applied on data acquired on a real scenario, which is more complex and is additionally affected by common measuring errors coming from real sensors.

#### 6.2.1. Segmenting a Virtual Scenario

**Figure 10:**
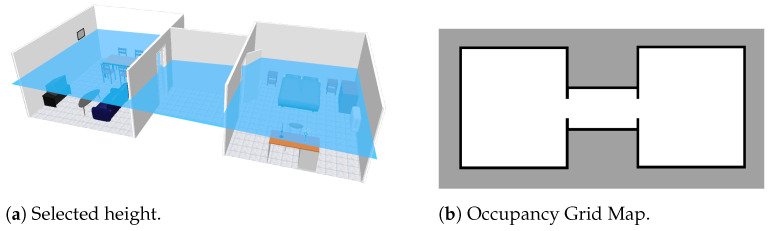
Creation of an occupancy grid map from point cloud data: (**a**) initial scenario with ceiling and frontal walls removed to allow the view of its interior, (**b**) selected height at which the point cloud data are collected, marked with a horizontal blue plane.

In the first experiment, a virtually created scenario is used to provide experimental results. The proposed scenario is comprised of two different rooms and a small corridor in between, counting a total of two doors. It contains several typical indoor objects such as tables or chairs, which could interfere with other possible segmentation algorithms. However, as it can be seen in Figure 10a, by extracting data at a certain height (marked with the horizontal blue plane), objects are avoided, so the final occupancy grid map (Figure 10b) is clearer.

**Figure 11:**
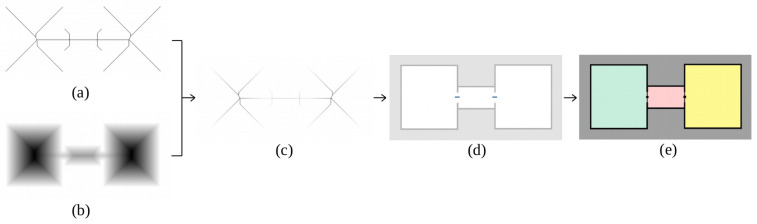
Geometric segmentation of occupancy grid maps using Voronoi diagrams: (**a**) skeleton, (**b**) distance transform, (**c**) combination of skeleton and distance transform, (**d**) selected segments according to standard door width, (**e**) segmentation results after including perpendicular line segments on every detected door.

The following steps are visually represented in Figure 11. Once the occupancy grid map is available at a certain height and after being processed to remove noise, both the skeleton (a) and the distance transform (b) of free space are extracted and multiplied together (c). Those pixels with values between 35 cm and 45 cm are selected, leaving small line segments (d). Central points of these lines are extracted and used to segment the scenario into rooms by perpendicularly drawing a line (e). As it can be seen, the two available doors are correctly detected and the scenario is correctly segmented into three different meaningful regions. Each of these regions is marked with a different color for better visualization of the results.

**Figure 12:**
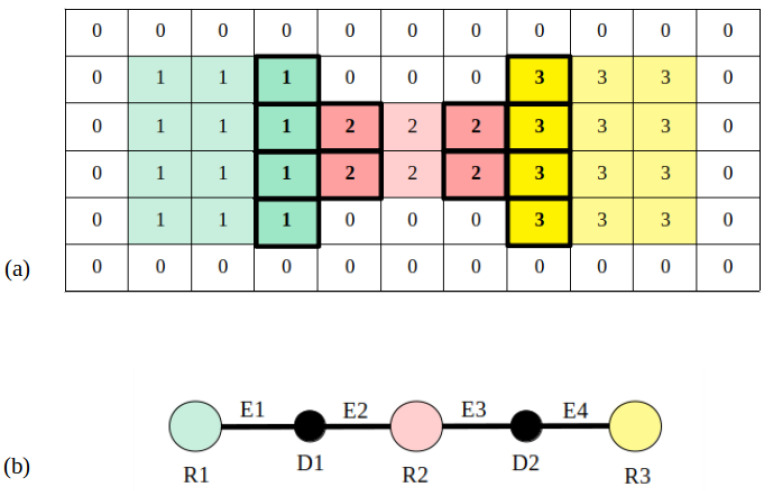
Topological map extraction from room labels: (**a**) Selection of pixels corresponding to doors (highlighted with a black rectangle), (**b**) final topological map, where a node is assigned to each room (R) and door (D), and edges (E) indicate connectivity among them.

Once the map is segmented, rooms are labeled (Figure 12a) and used to compute a topological structure. In this case, three different room nodes are extracted, each of them corresponding to a different segmented area. With respect to doors, two nodes are created and used to identify edges. Label numbers on each detected door are checked as follows. By looking at the left side of Figure 12a, labels 1 and 2 coincide on the same door, so two edges are created: the first one connecting room 1 to the door placed at the mentioned zone and the second one connecting room 2 to the door. The same is conducted for the right side of the image, using one edge to connect room 2 with the second door and another edge to connect room 3 with the second door. Results for the topological map are visually represented in Figure 12b.

#### 6.2.2. Overlapping Maps

**Figure 13:**
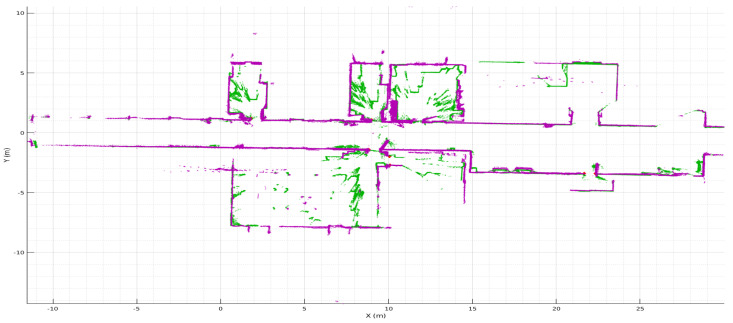
Overlapped maps.

Carrying on the implementations of all previous mapping techniques described before and to prove the main assumption of this research, Figure 13 shows the fused of two type of LiDAR data. Resulting overlapped maps helps to avoid or remove occlusions at different heights in indoor mapping, or even better, enhance room segmentation to further navigation.

After both maps where built using HS-SLAM, a last registration was needed to overlap both math. Green points represent the 2D map, where objects and occlusions at that level sparse points around zone areas. Purple map represent the cut made to the 3D map at 180 cm over the floor such as Figure 10a states, getting free rooms and virtually easier to segment at a geometrical level.

#### 6.2.3. Segmenting a Real Scenario

Once a simulated scenario has been used to provide a clearer approach to the proposed method, real data are used to prove the performance of the method in a more complex scenario using real data. The main challenge of this experiment is the presence of noise due to real measurements as well as a higher number of rooms and doors.

**Figure 14:**
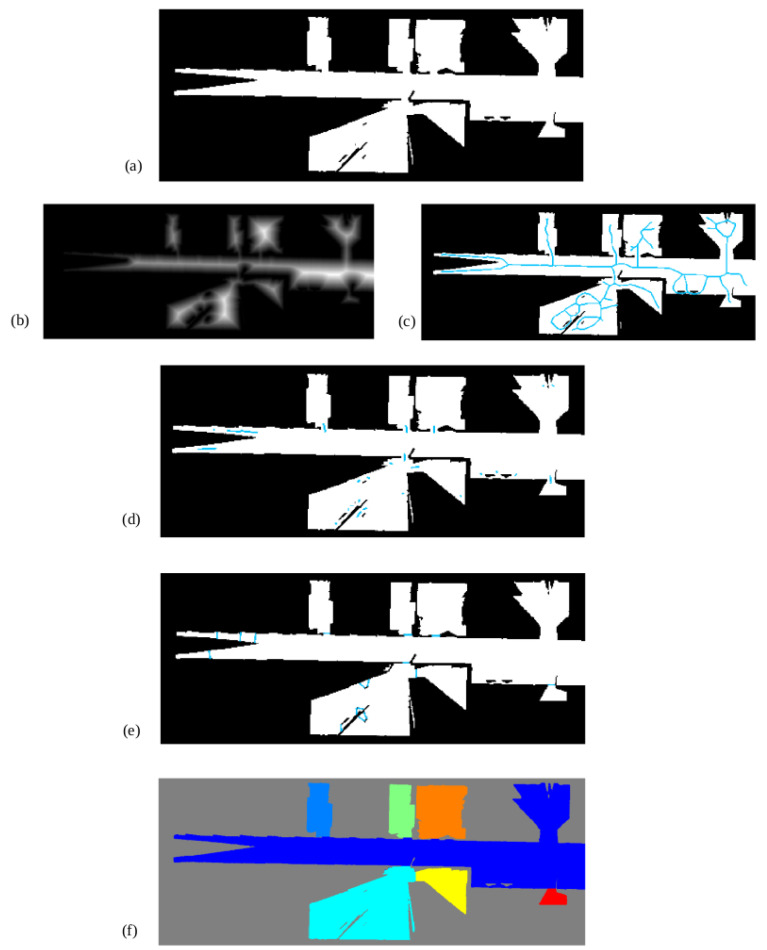
Results for the environment segmentation: (**a**) binary occupancy map, (**b**) distance transform of the free zones for the occupancy map, (**c**) skeleton marked in blue (**d**) line segments representing possible doors, (**e**) final geometric segmentation where areas smaller than 1.5 m${}^{2}$ have been merged with their neighbors, (**f**) rooms and corridors segmented with different colors.

The resulting binary occupancy map obtained from a slice of point cloud data at a certain height and preprocessed afterwards is shown in Figure 14a. There exists a noticeable difference between this representation and the one used on the previous experiment. Given that the scenario has bigger dimensions, some regions are only partially mapped. Additionally, some small occupied zones are included into free zones, which could interfere with the segmentation procedure.

**Figure 15:**
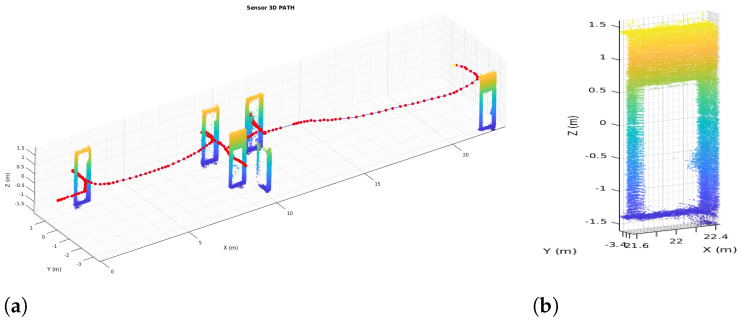
Door extraction. (**a**) SLAM path and Doors. (**b**) Single door.

Figure 14b,c show the extracted distance transform and Voronoi graph, respectively, which are combined and filtered using door width to derive into results shown in Figure 14d. Line segments are used to perpendicularly create map partitions (Figure 14e). As it can be seen, these partitions are correctly placed at doors. However, small partitions are created due to mapping errors. By removing zones under 1.5 m${}^{2}$ (a value way smaller that the typical room size), these errors are removed and the final map is correctly partitioned (Figure 14f). By getting all the doors detected and located in the 2D occupancy grid, a back propagation of locations is used to find and extract doors in the 2D and 3D geometric maps. Figure 15 shows the extracted elements. In Figure 15a all doors are extracted from the 3D map cloud, and the red line represents sensor path during mapping. Figure 15b shows a close-up view of the extraction and vertical cut of one door.

**Figure 16:**
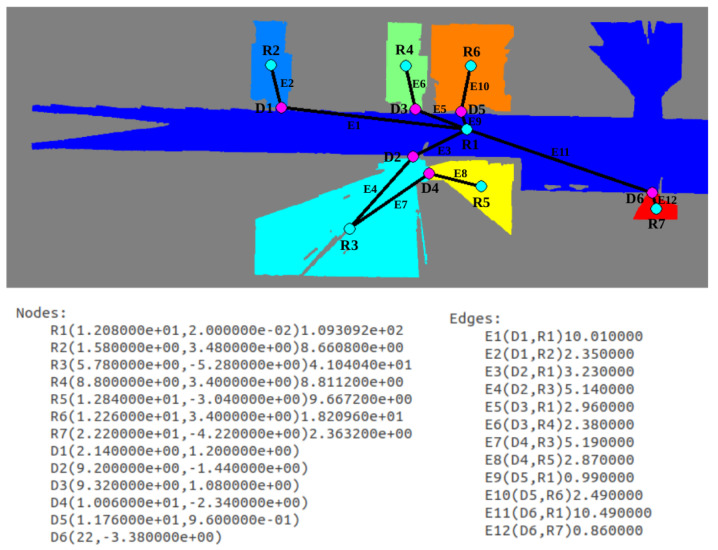
Resulting topological map from the segmented scenario. A node is assigned to every room and every door. Edges are derived by checking room connectivity at doors.

Finally, the topological map is extracted by assigning room nodes to each labeled zone, door nodes to each room separation and edges to represent connectivity between regions. Results are visually shown in Figure 16, where the resulting graph is overlapped with the labeled image and annotated for a better understanding. Below the map, the list of nodes and edges is provided. Nodes starting with an R correspond to rooms, and their centroid and area are saved in S.I. units. In the case of nodes starting with D, they correspond to doors are their centroid is saved. In the case of edges, first the two nodes that are connected by each edge is provided between brackets, and afterwards distance between the two node centroids is saved. In this way, a schematic representation of the environment is achieved, differentiating between the multiple zones of the indoor location and saving connectivity between them.

## 7. Discussion

**Figure 17:**
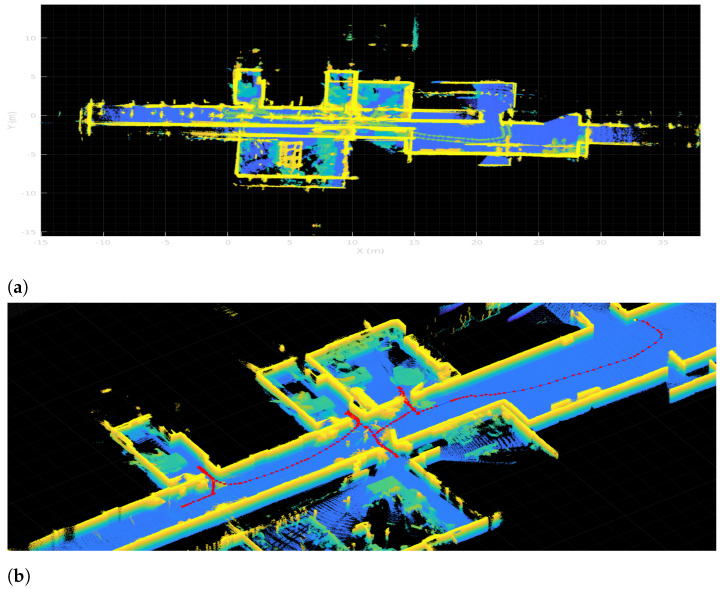
HS-SLAM vs. odometry. (**a**) 3D Robot Odometry map deformation. (**b**) 3D Lidar Odometry map corrected.

Concerning the steps made to achieved the goal of using and taking advanced of multi-LiDAR mapping and SLAM for scene segmentation in indoor environments, is necessary to compare the differences between maps before and after the simultaneous localization and mapping. Figure 2 against Figure 8 demonstrated an undoubtedly improve in map definition and correction, furthermore, Figure 17 displays the same benefits in its extension to 6D0F; in Figure 17a, the map matched using the robot odometry is misaligned, walls are thicker than they should be and rooms are not comprehensible nor well-defined, opposite, Figure 17b shows and prove the benefits of the HS-SLAM algorithm, the pose of the robot during mapping is corrected, map definition is better, walls are thinner, opened doors are well-defined and rooms are correctly aligned.

**Figure 18:**
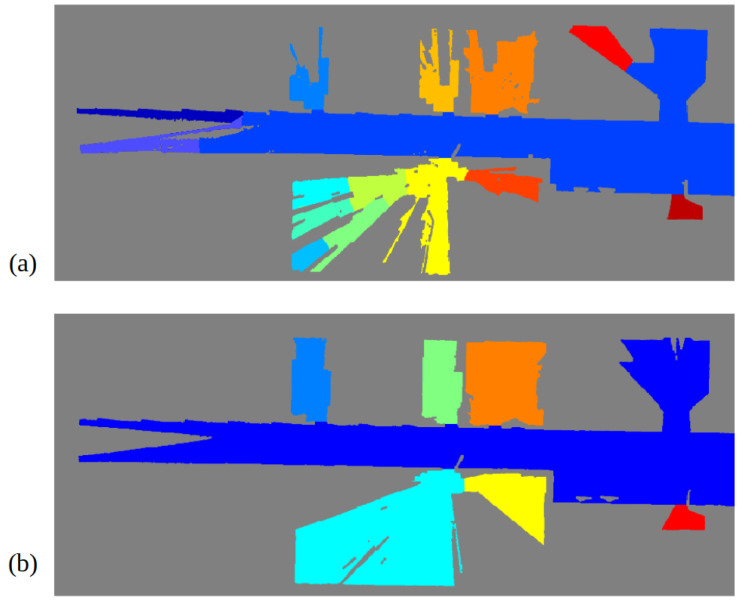
Segmentation results when the proposed algorithm is applied in two different maps: (**a**) floor-level map obtained from the Hokuyo laser, (**b**) derived map from a partitioning of 3D data from the Ouster LiDAR at a height of 1.7 m.

In order to prove the performance of the proposed segmentation procedure, a comparison with results using a traditional map at a floor level is provided. The same methodology depicted in Section 6.2.3 is applied on the map obtained from data gathered from the Hokuyo laser, placed at the robotic base. Results are shown in Figure 18, where map (a) is the one obtained from the Hokuyo data and map (b) is the one obtained from 3D data. Due to occlusions, map (a) does not correctly represent room shapes, leaving some parts as unknown since no data are available from these zones. This fact causes the creation of narrow spaces inside rooms with the same width as doors, interfering with the original doors and producing an oversegmentation of the space. The most representative example is the room coloured with light blue in map (b) (lower left room), which is divided into six different spaces in map (a), not matching the ground-truth segmentation. This fact proves the utility of using multiple sources of information for robotic applications, in this case room segmentation.

For a better appreciation of the method performance, precision and recall are measured according to [17]. Precision is an indicator of undersegmentation, which means that its value will be high if estimated rooms fit inside ground-truth rooms. On the contrary, recall is an indicator of oversegmentation, so it is high when ground-truth rooms fit inside estimated rooms. Both values need to be high for a good performance of the methods. Table 2 summarizes resulting precision and recall values for both the occluded and the non-occluded map. As it can be seen, both values are high for the non-occluded map, whereas in the case of the occluded one only precision is high. Given that recall does not even reach a 0.5, this value indicates that the scenario has been oversegmented, creating partitions where there are actually none.

As future work, it is intended to use the extracted maps to perform indoor navigation. With the use of Fast Marching Squared, a trajectory can be computed on the occupancy maps and executed by the robot, being capable of varying the original plan if an obstacle is detected during navigation. Furthermore, by checking door locations, the robot behaviour can be modified so it goes trough narrow passages in a safer manner. Additionally, future research aims object segmentation and scene recognition at semantic level, implementing 3D object extraction, multi objects relations and 3D space constraints for navigation, manipulation and grasp planning. The actual limitation of this work is the computational run-time for the HS-SLAM performance, so future works will aim to optimize algorithm computational time by studying possible parallelization techniques.

## 8. Conclusions

This section summarizes the goals accomplished during the research:

- Firstly, this research has introduced a new implementation of commonly used evolutive algorithms such as Harmony Search for scan matching algorithms and a working SLAM approach for both 2D and 3D environments. These have been tested on real-life applications on the ADAM robot for building a previous map for later use for path planning and scene segmentation. The precision of the Harmony scan registration is around 3 cm and less than 1.5${}^{\circ }$, performing well inside an expected range based on the sensors vertical and rotational accuracy and the uncertainty introduced by the fitness functions.
- Secondly, using the Hokuyo 2D LiDAR and the Ouster 3D LiDAR fused for different levels and height mapping proves its goal and solves the well-known occlusion problem that mostly takes place during a 2D mapping due to obstacles such as desks, chairs, and furniture.
- The application of a 2D occupancy grid both based on the Hokuyo LiDAR and a cut at a higher height of the 3D geometrical map based on the Ouster LiDAR improves significantly the door detection and segmentation on both point clouds level, boosting the chances of making a positive future path planning passing through halls, doors and rooms in occupancy grids with topological information.
- With the use of Voronoi diagrams extracted from free spaces at a certain height, a segmentation procedure has been carried out in which rooms have been efficiently differentiated by locating doors. The problem of over-segmentation due to occlusions in two-dimensional maps has been solved with the proposed approach by using geometric information at higher heights, proving the importance of using non-occluded maps. Recall has been improved with this method a 57.2%, from a value of 0.4838 to 0.7606.
- Finally, a topological map has been constructed by analyzing segmented rooms. In this way, a step is made toward how indoor scenarios are understood by people, using a higher abstraction level that is not mainly focused on specific geometric coordinates. Relevant information about the scenario shape and connectivity is schematically saved, being easy to handle for multiple applications such as topological planning or localization.

## Figures and Tables

**Table 1:** Scan matching precision.

	X	Y	Z	Rx	Ry	Rz
Max error	±3 cm	±5 cm	±0.1 cm	±0.1${}^{\circ }$	$0.1^{\circ }$	$1.5^{\circ }$

**Table 2:** Metrics for room segmentation.

	Occluded	Non-Occluded
Precision	0.8453	**0.8688**
Recall	0.4838	**0.7606**

## Abbreviations

The following abbreviations are used in this manuscript:

SLAM	Simultaneous Localization and Matching
DE	Differential Evolution
HS	Harmony Search
ICP	Iterative Closest Point
NDT	Normal Distribution Transform
HS-SLAM	Harmony Simultaneous Localization and Matching
